# Supplementary material for: Enhancing birth weight outcomes through improved antenatal care in West African countries: Evidence from propensity score matching analysis
Source: PLOS Glob Public Health. 2025 Nov 20;5(11):e0005367. doi: 10.1371/journal.pgph.0005367 (PMC12633929; doi:10.1371/journal.pgph.0005367)
Supplement: Attachment — Submitted filename: Response to reviewer.docx [file pgph.0005367.s003.docx]

**A point-by-point response to editor and reviewer**

**Authors’ Response to Academic Editor**

**Dear: Shiyam Sunder, PhD, Academic Editor**

We thank you for a thorough reading and constructive comments and suggestions on our manuscript and for the opportunity to revise and resubmit. We are pleased to submit the revised version of the manuscript titled “**“Enhancing Birth Weight Outcomes through Improved Antenatal Care in West African Countries: Evidence from Propensity Score Matching Analysis” (PGPH-D-25-02200)**. The comments from the editors and the reviewers were highly insightful and enabled us to greatly improve the quality of our manuscript. In this revised manuscript we made substantial changes to address your concerns in a point-by-point response. We appreciate your time and look forward to your response and we are very keen to incorporate further comments, if any, for the betterment of the final manuscript.

On the following pages, you will find our responses to the comments and suggestions raised by the esteemed reviewers 1 and 2.

Sincerely,

Aklilu Habte(MPH)(corresponding author)

[akliluhabte57@gmail.com](mailto:akliluhabte57@gmail.com)

**Authors’ response to Academic Editor**

**Comment 1.** Please amend your detailed Financial Disclosure statement. This is published with the article. It must therefore be completed in full sentences and contain the exact wording you wish to be published.

**Response:** Thank you for your valuable suggestion. We have made the correction and highlighted it in the ‘Funding’ section of the ‘Revised manuscript with track changes’ (Line 550, Page 26)

**Comment 2:** Regarding the data availability

**Response:** The data used in our study were obtained from the Demographic and Health Surveys (DHS) Program. These datasets are publicly available in anonymised form but require users to submit a reasonable request via the DHS website (<https://dhsprogram.com/Countries/> ) to ensure compliance with ethical and legal standards, including protection of participant confidentiality. Given that the DHS Program governs access to these datasets and requires registration and approval for use, we are unable to deposit the full dataset in a public repository or upload it as supplementary material. Public deposition would breach the terms of use and potentially conflict with the protocol approved by the DHS office.

We kindly request an exemption from the public data deposition requirement on these grounds.

**Comment 3:** If the reviewer comments include a recommendation to cite specific previously published works, please review and evaluate these publications to determine whether they are relevant and should be cited. There is no requirement to cite these works unless the editor has indicated otherwise.

**Response:** In our case, neither the reviewers nor the editors recommended citing any specific previously published works, so no additional citations were added.

**Comment 4:** regarding the use of retracted reference/s

**Response:** Thank you for the reminder. We have carefully reviewed all references cited in the manuscript and confirm that none of them have been retracted. Therefore, no changes were necessary to the reference list in this regard.

**Authors’ Response to Reviewer**#**1 (Emmanuel Ekung)**

**General comment:** The manuscript is timely and explicitly reports important public health issue (low birth weight), with minor corrections I recommend it for publication as this will inform public health interventions in maternal and child healthcare.

The following are the areas to be addressed by authors; -

Dear Reviewer 1, thank you very much for taking the time to review our work and for your positive feedback. We received your thoughtful, and generous review, along with helpful feedback and suggestions, as a valuable contribution to our ongoing work. We have tried to address all the possible comments and suggestions raised by you in the following session.

**Comment 1:** The manuscript addresses a highly relevant public health issue (low birth weight in West Africa) using multi-country DHS data and propensity score matching (PSM), which is methodologically sound and ethically appropriate for secondary data use.

However: The framing in the Introduction could be more concise and explicitly linked to equity and global health relevance, as required by the journal.

**Response:** Thank you for your thoughtful feedback. We appreciate your recognition of the methodological and ethical strengths of our study. In response to your suggestion, we have revised the Introduction to improve clarity and conciseness, and to more explicitly frame the issue of low birth weight within the context of equity and global health relevance. The newly added statements are highlighted in the Introduction section of the revised manuscript with track changes (Lines 98–100 and 108-111, Page 4).

**Comment 2:** Result sections are overly detailed with large tables that may overwhelm readers; clarity and focus on the most important findings would improve accessibility.

**Response:** Thank you for the constructive feedback. We acknowledge that the Results section was relatively detailed, as our intention was to transparently present the associations of each attribute with both treatment groups (ANC4+ and ANC8+) as well as the outcome of interest (LBW). In response to your suggestion, we have revised the section to enhance clarity and focus, streamlining both the narrative and tables to emphasize the most salient findings and improve readability. Additionally, the longest table (Table 1) has been integrated into Tables 3 and 4 by adding a column that presents the total weighted prevalence of each attribute. These updates are clearly highlighted in Tables 3 and 4 of the revised manuscript with track changes (Page 10 and 12).

**Comment 3:** The statistical analysis been performed appropriately and rigorously. The use of PSM is appropriate for causal inference in observational DHS data. Matching diagnostics, sensitivity analyses (Rosenbaum bounds), and balance checks are clearly presented and robust. The statistical approach supports the conclusions. Minor refinements may include the following: -

- Explicit mention of how missing data were handled.
- A clearer justification for choosing caliper = 0.01 in radius matching.

**Response:** Thank you for your positive assessment of our statistical analysis and for the helpful suggestions regarding minor refinements. We have now addressed the following points:

- Missing Data: Observations with missing values on the treatment variables (ANC uptake) and the outcome variable (birthweight) were excluded prior to the start of the analysis to ensure consistency and validity in the propensity score matching process.
- Caliper Justification: We selected a caliper of 0.01 for radius matching to ensure a high degree of similarity between matched units while retaining sufficient sample size. This threshold reflects a conservative balance between minimizing bias and maximizing matching efficiency, and is consistent with established practices in similar DHS-based studies.

All these were highlighted in the Method section of the revised manuscript.

**Comment 4:** The authors have not made all data underlying the findings fully available

The manuscript states that DHS data were used and available at https://dhsprogram.com. However, the Data Availability Statement does not currently comply with PLOS requirements: Authors must provide a direct DOI or persistent URL to the DHS datasets used, specifying country, year, and dataset type (IR file). A statement such as: “ The data are publicly available upon registration from the DHS Program (https://dhsprogram.com). The specific datasets used in this study include (list of 14 country-years, IR files).” should be added.

**Response:** Thank you for the helpful suggestion. We have updated the Data Availability Statement to comply with PLOS requirements by including a valid URL that directly links to the specific DHS country datasets used in our analysis. The revised statement now specifies the country, year, and dataset type (IR files), and clearly indicates that the data are publicly available upon registration from the DHS Program. These changes are highlighted in the ‘Data Availability’ section of the ‘revised manuscript with track changes’ (Line 545-550, Page 23).

**Comment 5:** The manuscript is generally understandable but requires language polishing for clarity and conciseness. There are grammatical inconsistencies (e.g., “Of the weighted sample, 71.38% and 14.54% of women received ANC4+ and ANC8+, respectively.

**Response:** Thank you for the helpful feedback regarding language clarity and grammatical consistency. We have thoroughly reviewed the manuscript to correct grammatical errors and improve overall conciseness. Specifically, the statement regarding the magnitude of ANC4+ and ANC8+ coverage has been revised for clarity and is now highlighted in the ‘Results’ section of the ‘Revised manuscript with track changes’ (Lines 299–301, Page 13).

**Comment 6:** Some sections are repetitive (e.g., ANC definitions repeated in Introduction, Methods, and Results). Long tables could be simplified or moved to supplementary materials, with summary results in the main text, to enhance readability and comprehension.

**Response:** Thank you for your insightful feedback. We have carefully considered your suggestions throughout the revised manuscript. To reduce redundancy, we streamlined the presentation of ANC definitions and avoided repetition across the Introduction, Methods, and Results sections. Additionally, we simplified the longer tables and, where appropriate, moved detailed content to the supplementary materials. Summary results are now emphasized in the main text to enhance readability and overall comprehension.

**Comment 7:** The Discussion does not sufficiently contextualize findings within regional health policy frameworks or highlight implications for equity, health systems, and vulnerable populations.

**Response:** Thank you for the valuable feedback. We acknowledge that the Discussion section could more clearly situate the findings within relevant regional health policy frameworks. To strengthen this, we will incorporate references to current policy initiatives and strategies that shape healthcare delivery in the region. Additionally, we will expand the discussion to explicitly address implications for health equity, system-level impacts, and considerations for vulnerable populations—particularly in terms of access, resource allocation, and long-term sustainability. These additions will help ensure the findings are not only interpreted in context but also aligned with broader public health priorities. The additions are highlighted throughout the ’Discussion’ section of the revised manuscript with track changes.

Thank you for your constructive comments and suggestions, which we got as valuable input in the improvement of our manuscript. We received all of them as a valuable contribution to our ongoing work.

**_________________________________________________________________________________________________________END_______________________________________**

**THANK YOU!!!**

**Authors’ response to reviewer #2**

To the editorial team, thank you for the opportunity to review this manuscript. This is an important research piece that has potentially a high impact on maternal and child health. However, I have a few comments that can help make it stronger.

**Response:** Dear Reviewer 2, thank you very much for taking the time to review our work and for your positive feedback. We received your thoughtful, and generous review, along with helpful feedback and suggestions, as a valuable contribution to our ongoing work. We have tried to address all the possible comments and suggestions raised by you in the following session.

**Comment 1:** in the methods sections, the selection of the countries and the specific years in review should be explained methodically, or in case of convenience, should be indicated.

**Response:** Thank you for the insightful observation. For this analysis, we included 14 countries from the West African region based on the availability of recent and comprehensive survey data. Specifically, we considered countries that had conducted Demographic and Health Surveys (DHS) from 2010 onward, and we got countries with surveys conducted between 2012 and 2023. This selection was partly guided by convenience, as these datasets provided the most complete information on antenatal care utilization and birth outcomes relevant to our study objectives. The final weighted sample comprised 51,455 eligible women, representing a robust cross-section of maternal health experiences across the region. This amended statement is highlighted in the ‘Study design, period, and population’ section of the ‘Revised Manuscript with track changes’ (Line 124-127, Page 4).

**Comment 2:** the use of the codes- 9996, 9997, etc, should be explained in more detailed.

**Response:** Thank you for the helpful comment. We will revise the Methods section to provide a clearer explanation of the codes 9996, 9997, and similar entries. These codes are standard placeholders used in the dataset to indicate specific types of missing or non-applicable responses. The revision was highlighted in the ‘Outcome variable’ section of the ‘Revised Manuscript with track changes’ (Line145-146, Page 5).

**Comment 3:** Since the data is from DHS - a secondary data, I am more concerned with data quality issues rather than recall bias and social desirability effect. It will be helpful to explain how that could be a concerned, in the limitation section.

**Response:** Thank you for the thoughtful comment. While we acknowledge that data quality is a central concern when working with secondary sources like DHS, we also recognize that the cross-sectional nature of the study and the use of face-to-face interviews introduce inherent limitations such as recall bias and social desirability effects. These biases are particularly relevant in self-reported measures, including antenatal care visits and birth outcomes. That is why we explicitly included them in the limitations section—to reflect the broader context of data collection and interpretation challenges associated with demographic survey data.

**Comment 4:** This is actually a well written manuscript; however, I will encourage a review for typos/grammar. In the results section, please correct the typos in the first paragraph

**Response:** Thank you for the encouraging feedback. We appreciate your positive remarks on the manuscript's overall quality. We will carefully review the entire manuscript to correct any typos and grammatical issues, ensuring clarity and consistency throughout the text. Your suggestion is well taken and will help improve the readability of the manuscript.

**________________________________________________________________________________________________________END_______________________________________**

**THANK YOU!!!**
